# Termite Diversity in Urban Landscape, South Jakarta, Indonesia

**DOI:** 10.3390/insects7020020

**Published:** 2016-05-06

**Authors:** Arinana, Rifat Aldina, Dodi Nandika, Aunu Rauf, Idham S. Harahap, I Made Sumertajaya, Effendi Tri Bahtiar

**Affiliations:** 1 Faculty of Forestry, Bogor Agricultural University, Bogor 16680, West Java, Indonesia; rifataldina@gmail.com (R.A.); nandikadodi@gmail.com (D.N.); bahtiar_et@yahoo.com (E.T.B.); 2 Faculty of Agriculture, Bogor Agricultural University, Bogor 16680, West Java, Indonesia; aunurauf@gmail.com (A.R.); idham@biotrop.org (I.S.H.); 3 Faculty of Mathematics and Natural Sciences, Bogor Agricultural University, Bogor 16680, West Java, Indonesia; imsjaya@yahoo.com

**Keywords:** termite dispersion region, termite diversity and distribution, termite identification, soil characteristics and local weather effect

## Abstract

The population of South Jakarta, a city within the Province of Jakarta Capital Region, is increasing annually, and the development of land into building causes termite diversity loss. The aim of this research was to determine the diversity of subterranean termite species and their distribution in South Jakarta and to evaluate the soil profile termite habitat. This study was conducted in South Jakarta and was carried out at four residential areas representing four randomly selected sub-districts. Specimens were collected with a baiting system. At each residence, as many as 25–30 stakes of pine wood (*Pinus merkusii*) sized 2 cm × 2 cm × 46 cm were placed for termite sampling. Soil samples were also collected from each residence for testing of their texture, pH, soil water content, and C-organic. Three species of subterranean termites were identified, including *Coptotermes curvignathus*, *Microtermes insperatus*, and *Macrotermes gilvus*, with area-specific variations in occurrence. The soil and weather conditions in the studied areas provided suitable habitat for termites, and *M. insperatus* was the most commonly found species.

## 1. Introduction

Termites frequently destroy wood as well as other cellulose materials used in building construction. The insects have successfully adapted to the varied conditions that characterize urban and agricultural environments around the world [1], and their attacks on buildings are an important issue. The intensity of these attacks is high and ongoing, and economic losses associated with them tend to increase from year to year. Subterranean termites are the most commonly occurring type, and they are potentially the most destructive pest in any building. The intensity of a termite attack and the magnitude of the resulting damage can be very high. According to Nandika *et al.* [2], economic losses from termite-related damage in residential buildings in Indonesia was estimated to be Rp 8.7 trillion in 2015. The threat of termite attack on buildings will remain high.

The climate of South Jakarta features moderate rain intensity, and the humid weather can cause building damage, such as cracks and decay. This damage can provide access for termites to enter the buildings. Termites not only attack the cellulose-containing components of a building, such as window or door frames, doors, roofs, and ceilings, but also the interior wooden structures, such as furniture and sculpture. This attack will certainly inflict a high economic cost on maintaining buildings. The results of the current research study are expected to provide information about the diversity and distribution of subterranean termite species in residential areas and to identify the intensity of attacks in residential buildings.

## 2. Experimental Section

### 2.1. Time and Locations

This research was conducted from December 2014 to April 2015 in four residences chosen from four randomly selected sub-districts out of 10 in South Jakarta: Tebet Barat (latitude: 6°14′03′′S, longitude: 106°50′60′′E, altitude 17 m.a.s.l.), Jagakarsa (06°19′31′′S, 106°49′06′′E, 62 m.a.s.l.), Pasar Minggu (06°17′38′′S, 106°49′25′′E, 39 m.a.s.l.), and Cipulir (06°14′13′′S, 106°46′25′′E, 24 m.a.s.l.). Identification of termites was conducted at the Laboratory of Wood Quality Improvement Technology, Department of Forest Products, Faculty of Forestry, Bogor Agricultural University (IPB). Soil analysis was carried out at the Laboratory of Integrated Soil Resource, Soil Research Institute, Agency for Agricultural Research and Development, Ministry of Agriculture, Bogor.

### 2.2. Equipment and Materials

The equipment used in this research included bottles for termite collection, brushes, a digital camera, a stereo microscope, environment meter (thermo-hygro and lux meter) (Krisbow), transparent plastic, surgical instruments for termite dissection, and a soil driller. The materials used were air-dried pine wood (*Pinus merkusii*) stakes measuring 2 cm × 2 cm × 46 cm, oil paint (red color), and alcohol 70%.

### 2.3. Collection and Identification of Termite Species

Pine wood was chosen for the stakes because Arinana *et al.* [3] found that subterranean termites prefer pine wood to *Acacia mangium*, *Hevea brasiliensis*, and *Paraserianthes falcataria*. The tops of the stakes were painted with a bright color (red) to help mark their location. The size of the stakes used followed the ASTM standard [4].

**Figure 1:**
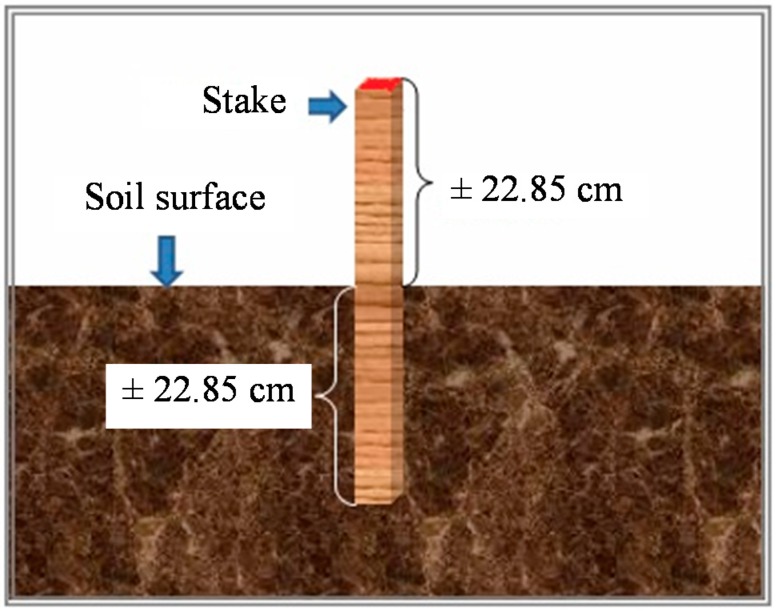
Installation of stakes.

Termites were collected with a baiting system, which included 108 stakes in all investigated areas (25–30 stakes per residence). The stakes were installed around the house and outdoor structures on the property in areas that were not covered by concrete or other artificial barriers. Some stakes were also installed in outdoor community areas such as playgrounds, gardens, parks, *etc*. Stakes amounted to as many as 25, 28, 30, and 25 pieces, spread around the housing areas in Tebet Barat, Jagakarsa, Pasar Minggu, and Cipulir, respectively. Stakes were installed vertically into the soil so that half was below the soil surface, as shown in Figure 1. Stakes were left in place for 3 months and checked monthly for termite infestation.

Stakes that were attacked by termites were extracted, and soldiers were collected and preserved in 70% alcohol. The species of the soldier termites were then identified based on literature and key identification according to Ahmad [5] and Tho [6]. The collected termites were photographed and observed by microscope under 10× magnification for the entire insect body and 30× magnification for the head parts including the antennae and mandibles.

### 2.4. Soil Characteristics Analysis

Soil sampling was conducted at four points (Eastern, Western, Southern, and Northern parts) in every region of the study at depths of 0–20 cm and 20–40 cm; thus, there were 32 soil samples in total. The soil samples were sent to the Laboratory of Integrated Soil Resource (an ISO/IEC1705 certified laboratory) to measure the soil moisture content, pH, C-organic content, and texture.

### 2.5. Sunlight Intensity, Temperature, and Humidity Measurements

Measurements of sunlight intensity, temperature, and humidity were conducted in the morning (8:00 a.m.), noon (12:00 a.m.), and afternoon (16:00 p.m.).

## 3. Results and Discussion

### 3.1. Diversity of Subterranean Termites

Twenty-one stakes were attacked during the 3 months those stakes were in place, and the identification and method of determination based on Ahmad [5] and Tho [6] revealed that three species of subterranean termites were present. The termites were members of two termite families, namely Rhinotermitidae and Termitidae. The diversity of subterranean termites in South Jakarta is shown in Table 1.

**Figure 2:**
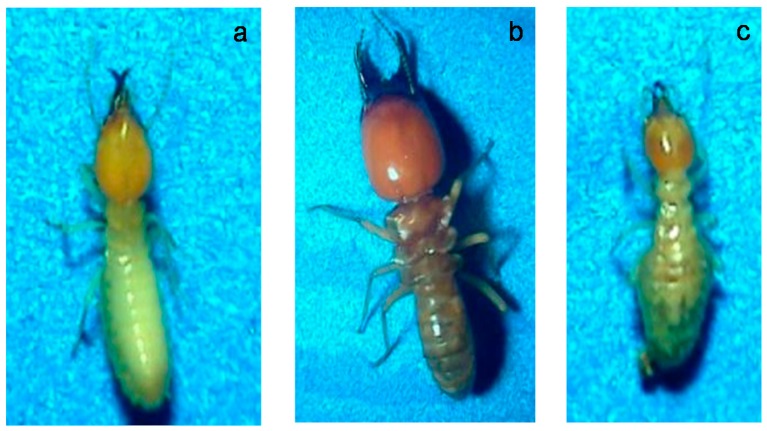
Termites found at the research locations: (**a**) *C. curvignathus*; (**b**) *M. gilvus*; (**c**) *M. insperatus*.

The species found in the research area were *Microtermes insperatus* (attacked sixteen stakes), *Macrotermes gilvus* (attacked three stakes), and *Coptotermes curvignathus* (attacked two stakes). Nandika [2] who conducted similar research in West Jakarta and East Jakarta also reported that *M. insperatus* was frequently found in those two areas. Meanwhile Arinana *et al.* [7] reported that *C. curvignathus* was dominant in residences of Bumi Bekasi-West Java (a nearby district to Jakarta). Figure 2 shows representative photographs of soldier termites found in the research areas. More complete identification information about the termites that attacked the wood stake bait are shown in the appendix.

**Figure 3:**
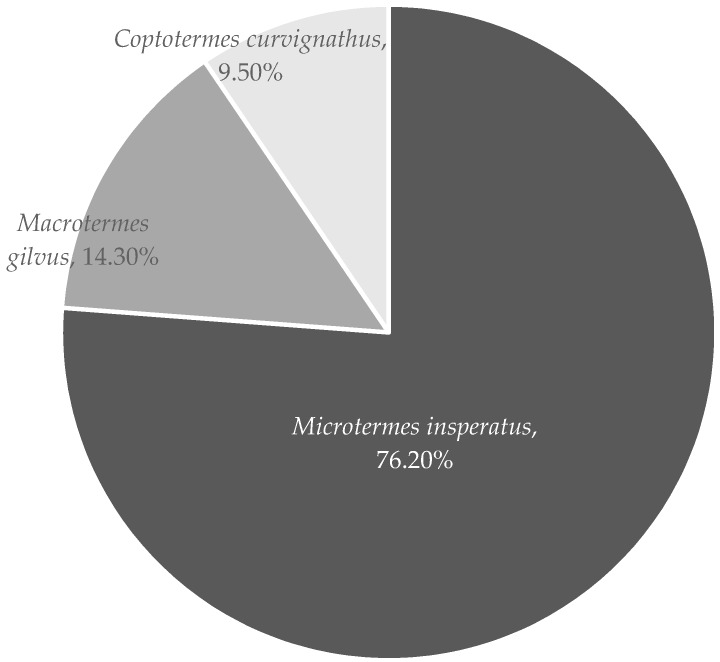
Percentages of subterranean termites represented in South Jakarta.

The results of this research showed that the Termitidae (90.48%) family was more dominant than the Rhinotermitidae (9.52%). Eggleton [8] reported that the Termitidae family was dominant in the tropical region. According to Wang *et al.* [9], species of Rhinotermitidae are found more frequently outside natural forests or in areas of natural forest that have been converted into plantations, estates, or farms. The subfamilies of Rhinotermitidae found in the research were Coptotermitinae (*C. curvignathus*), members of the family Rhinotermitidae, genus *Coptotermes*, which are the most likely to attack wood and buildings [10]. Meanwhile, the subfamilies of Termitidae found in the research area was Macrotermitinae, with members *M. gilvus* and *M. insperatus.* Of the three species found, the wood-destroying capabilities of *C. curvignathus* were the highest. *C. curvignathus* has been reported to attack up to the top of a high rise apartment building in South Jakarta [11]. The percentages of termite species are provided in Figure 3.

### 3.2. Soil Characteristics

The soil at each residence contained high amounts of clay and C-organic. Termites can increase the organic matter content in soil that is used to build a nest, and they also modify the composition of clay minerals [12]. Soil characteristics are shown in Table 2.

Ground water levels regulate the levels of air and gas exchange that affect the soil properties (water content, pH, texture) and activity of organisms in the soil. There were strong correlations between termite infestation and soil water content and texture (Table 3). Our results showed a neutral pH range between 6.36 and 7.30 and a water content ranging from 22.10% to 31.07%. In those ranges of pH and soil water content, the termites attacked more frequently in the higher moisture content than the lower one. Hillel [13] stated that many other soil properties depend on moisture content, including mechanical properties such as consistency, plasticity, and strength. The characteristics of clay, soil shrinkage, and development are related to the addition or subtraction of water, which causes changes in the density, porosity, and pore size distribution. Termites prefer to nest in silty clay rather than in the loamy sand of soil.

White [14] argues that the highest C-organic content is located at a depth of 15–20 cm below the soil surface. This is consistent with our research showing that levels of C-organic ranged from 5.44% to 9.55%. C-organic content in 0–20 cm depth of soil has a positive correlation with termite infestation level (Table 3). According to Lee and Wood [15], land with termite activity has very high organic matter content. This agrees with the work of Foth [16], who stated that organic material is influenced by the current amount of the original material and by the rate of decomposition and humification, which are highly dependent on environmental conditions.

### 3.3. Sunlight Intensity, Temperature, and Humidity Measurements

Termite colonies are usually maintained in constant temperature (25–36 °C) and high humidity. Termites build nests and hives to protect the microclimate habitat from outer environment interference. The temperature in the research location showed an optimum temperature range for subterranean termites’ life, with a minimum temperature of 26.2 °C and a maximum of 34.7 °C; meanwhile, the atmospheric humidity was considered too low for termite life (Table 4). The lowest humidity (54.5%) in the investigated areas occurred during the day, and the highest humidity (79.4%) was in the morning. According to Nandika *et al.* [17], a suitable humidity for termites ranges between 75% and 90%. The termite nests and hives were built to maintain humidity levels so that the thin-skinned workers can be protected from drying out. Termites are able to create a microclimate inside the hive, however, and humidity levels found in this study are still tolerable.

Since the external temperature of all investigated areas were suitable enough for termite life, the correlation of external temperature and termite infestation was low; meanwhile, the sunlight intensity and humidity were strongly correlated to termite infestation (Table 5). Bahtiar *et al.* [18] reported that sunlight intensity directly and strongly correlated to the temperature and humidity. The temperature and humidity fluctuation is mainly following the sunlight intensity in the area. The sunlight generates radiation and increases the earth’s surface energy, which causes the variation in temperature and humidity. The higher sunlight intensity means higher temperature and lower humidity in the investigated area. Since it has a strong correlation with humidity, sunlight intensity was also significantly related to the termite infestation level. Sunlight intensity is also related to swarmer activity which start the termite colony spread in the area. This phenomenon confirmed the higher frequency of termite infestation in the areas with higher sunlight intensity. Similar results have been reported by Axelsson an Anderson [19], who found that average termite mound height had a positive correlation with the average percentage of light. The reduction of canopy cover would increase the sunlight intensity and initiate more intense termite activity.

## 4. Conclusions

Three species of subterranean termites, namely *Microtermes insperatus*, *Macrotermes gilvus*, and *Coptotermes curvignathus*, were found in the urban landscape of South Jakarta. *M. insperatus* occurs with the highest frequency, followed by the other species. The soil and climate characteristics in the entire region provided a habitat conducive to subterranean termites.

## Figures and Tables

**Table 1:** Diversity of subterranean termites from four residences in South Jakarta.

Residential	Number of Installed Stakes	Percentage of Infested Stakes	Stakes Code	Family	Termite Species
Tebet Barat	25	8.0%	A1	Rhinotermitidae	*Coptotermes curvignathus*
			A2	Rhinotermitidae	*C. curvignathus*
Jagakarsa	28	21.4%	B1	Termitidae	*Microtermes insperatus*
			B2	Termitidae	*M. insperatus*
			B3	Termitidae	*Macrotermes gilvus*
			B4	Termitidae	*M. insperatus*
			B5	Termitidae	*M. insperatus*
			B6	Termitidae	*M. gilvus*
Pasar Minggu	30	36.7%	C1	Termitidae	*M. insperatus*
			C2	Termitidae	*M. gilvus*
			C3	Termitidae	*M. insperatus*
			C4	Termitidae	*M. insperatus*
			C5	Termitidae	*M. insperatus*
			C6	Termitidae	*M. insperatus*
			C7	Termitidae	*M. insperatus*
			C8	Termitidae	*M. insperatus*
			C9	Termitidae	*M. insperatus*
			C10	Termitidae	*M. insperatus*
			C11	Termitidae	*M. insperatus*
Cipulir	25	8.0%	D1	Termitidae	*M. insperatus*
			D2	Termitidae	*M. insperatus*

**Table 2:** Soil characteristics at four residences in South Jakarta.

Residential	Depth (cm)	Water Content (%)	pH	C-Organic (%)	Sand (%)	Silt (%)	Clay (%)	Soil Criteria
Tebet Barat	0–20	27.42	6.85	6.66	46	35	19	Loam
	20–40	22.10	7.30	5.44	19	35	46	Clay
Jagakarsa	0–20	31.07	6.68	9.55	11	33	56	Clay
	20–40	30.20	6.90	8.61	12	26	62	Clay
Pasar Minggu	0–20	28.77	6.72	7.52	5	55	40	Silty Clay
	20–40	23.48	6.48	7.01	15	37	48	Clay
Cipulir	0–20	26.10	6.36	7.17	8	41	51	Silty Clay
	20–40	26.47	6.70	7.09	11	73	16	Loamy Sand

**Table 3:** Correlation between soil characteristics at a depth of 0–20 cm below the soil surface and percentage of termite infestation.

	Soil Water Content (%) *	pH *	C-Organic (%) *	Sand (%) *	Silt (%) *	Clay (%) *	Soil Criteria **
Coefficient of correlation (*r*)	0.564	0.270	0.359	−0.568	0.722	0.226	0.500
*p*-value	0.436	0.730	0.641	0.432	0.278	0.774	0.500

Note: * = parametric correlation; ** = non-parametric (Spearman) correlation.

**Table 4:** Measurement data of temperature, light intensity, and humidity at four residences in South Jakarta.

Residential	Time	Sunlight Intensity (lux)	Temperature (°C)	Humidity (%)
Tebet Barat	08.00	44,900	28.7	79.4
	12.00	64,700	32.7	63.4
	16.00	24,400	27.4	57.7
Jagakarsa	08.00	26,800	31.4	72.6
	12.00	57,900	29.7	60.1
	16.00	43,400	33.2	57.9
Pasar Minggu	08.00	64,800	26.2	71.6
	12.00	62,300	34.7	55.9
	16.00	26,100	32.5	61.2
Cipulir	08.00	29,900	31.5	71.8
	12.00	77,900	33.9	54.5
	16.00	23,000	32.7	67.1

**Table 5:** Correlation between weather characteristics and percentage of termite infestation.

	Sunlight Intensity (lux)	Temperature (°C)	Humidity (%)
Coefficient of correlation (*r*)	0.7832	0.0100	−0.7935
*p*-value	0.217	0.990	0.207

## Appendix

**Table A1:** The identification results of termites that attacked wood bait in South Jakarta.

Stake Code	Termite Species and Their Characteristics	Morphology
A1	*Coptotermes curvignathus* Characteristics: Average length of head with mandible: 1.77 mm Average length of head without mandible: 1.2 mm Width of head: 1.04 mm Overall length: 4.29 mm	
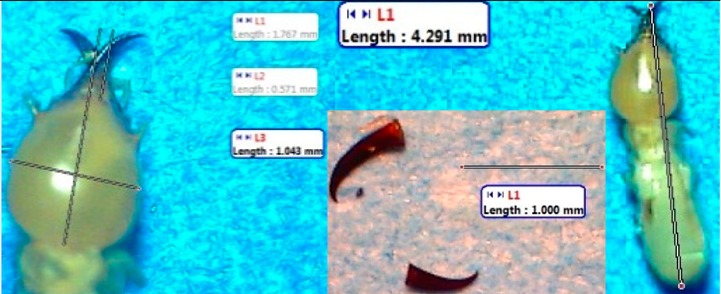
A2	*Coptotermes curvignathus* Characteristics: Average length of head with mandible: 1.96 mm Average length of head without mandible: 1.31 mm Width of head: 1.13 mm Overall length: 4.99 mm	
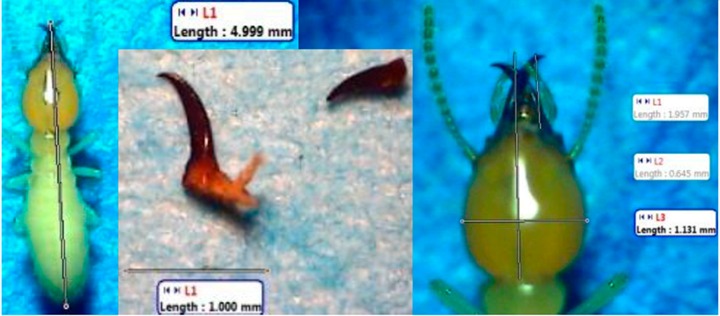
B1	*Microtermes insperatus* Characteristics: Average length of head with mandible: 1.6 mm Average length of head without mandible: 1.18 mm Width of head: 0.99 mm Overall length: 4.5 mm	
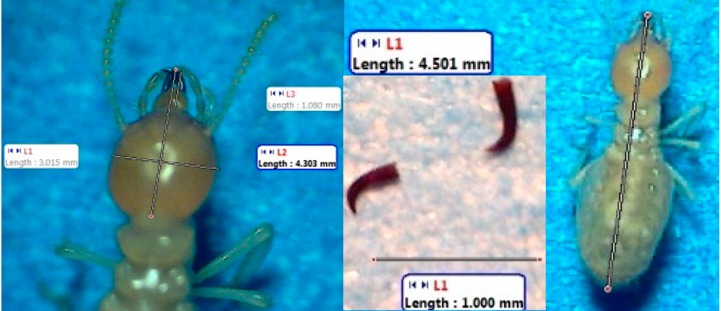
B2	*Microtermes insperatus* Characteristics: Average length of head with mandible: 1.28 mm Average length of head without mandible: 0.94 mm Width of head: 0.82 mm Overall length: 3.75 mm	
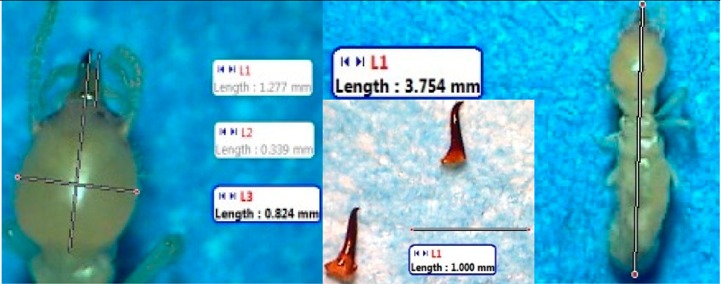
B3	*Macrotermes gilvus* Characteristics: Average length of head with mandible: 4.77 mm Average length of head without mandible: 3.0 mm Width of head: 2.73 mm Overall length: 10.8 mm	
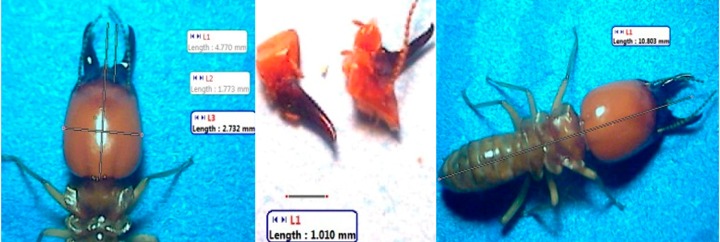
B4	*Microtermes insperatus* Characteristics: Average length of head with mandible: 1.35 mm Average length of head without mandible: 0.96 mm Width of head: 0.96 mm Overall length: 4.1 mm	
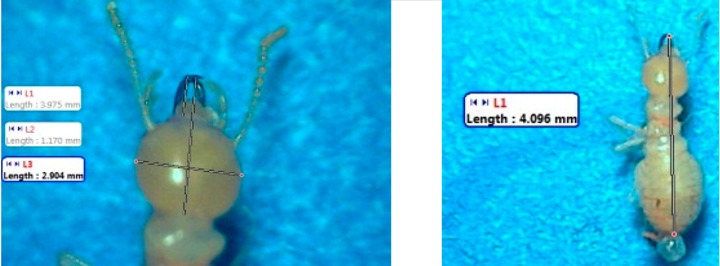
B5	*Microtermes insperatus* Characteristics: Average length of head with mandible: 1.41 mm Average length of head without mandible: 1.07 mm Width of head: 0.99 mm Overall length: 5.36 mm	
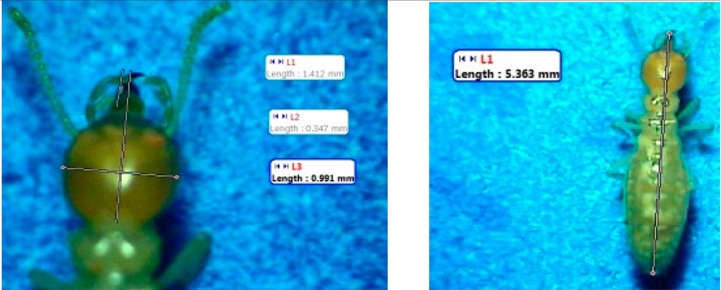
B6	*Macrotermes gilvus* Characteristics: Average length of head with mandible: 2.46 mm Average length of head without mandible: 1.37 mm Width of head: 1.58 mm Overall length: 6.8 mm	
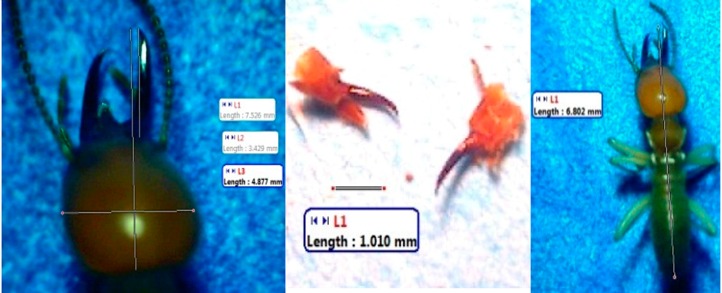
C1	*Microtermes insperatus* Characteristics: Average length of head with mandible: 1.6 mm Average length of head without mandible: 1.17 mm Width of head: 0.95 mm Overall length: 4.77 mm	
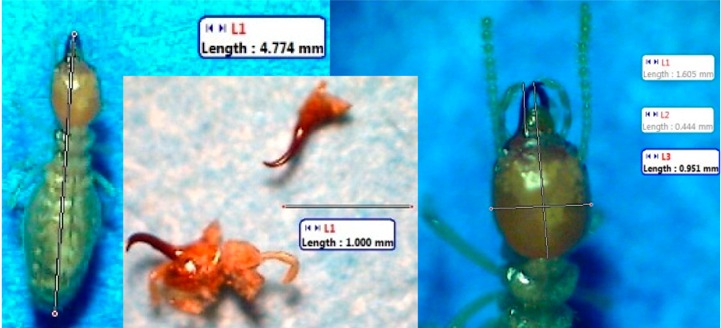
C2	*Macrotermes gilvus* Characteristics: Average length of head with mandible: 3.98 mm Average length of head without mandible: 2.55 mm Width of head: 2.57 mm Overall length: 8.31 mm	
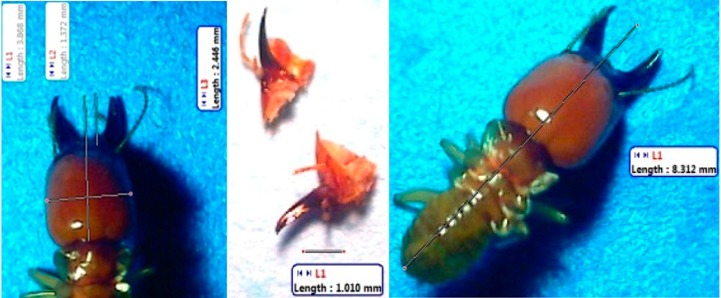
C3	*Microtermes insperatus* Characteristics: Average length of head with mandible: 1.43 mm Average length of head without mandible: 0.98 mm Width of head: 0.92 mm Overall length: 4.39 mm	
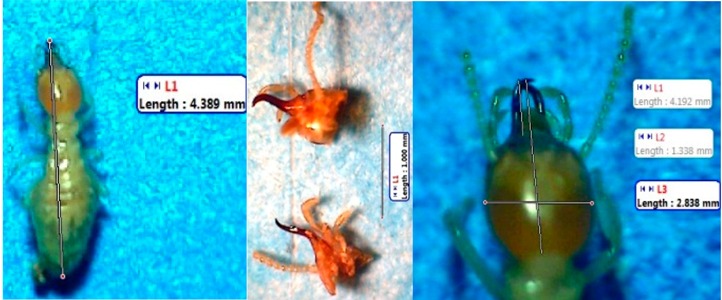
C4	*Microtermes insperatus* Characteristics: Average length of head with mandible: 1.5 mm Average length of head without mandible: 1.07 mm Width of head: 0.89 mm Overall length: 5.3 mm	
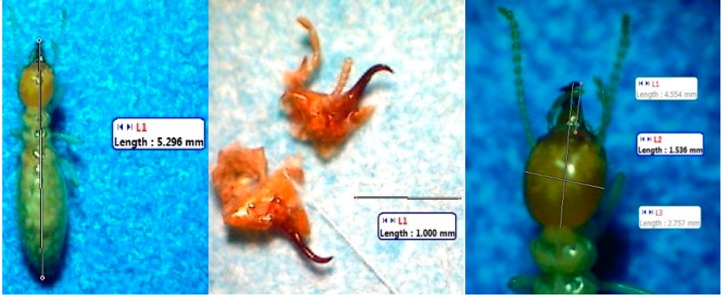
C5	*Microtermes insperatus* Characteristics: Average length of head with mandible: 1.45 mm Average length of head without mandible: 1.09 mm Width of head: 1.0 mm Overall length: 5.51 mm	
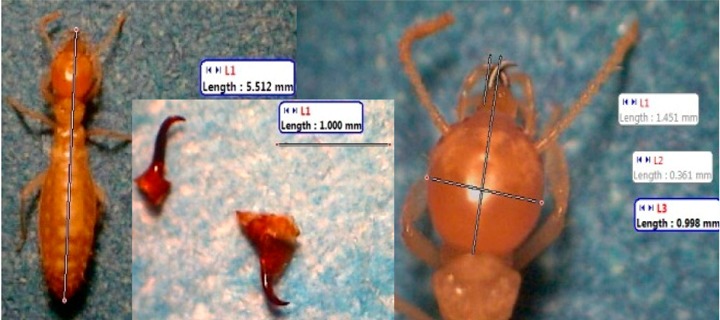
C6	*Microtermes insperatus* Characteristics: Average length of head with mandible: 1.29 mm Average length of head without mandible: 0.88 mm Width of head: 0.79 mm Overall length: 4.26 mm	
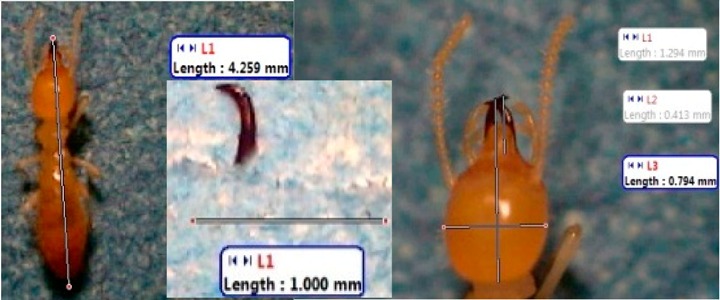
C7	*Microtermes insperatus* Characteristics: Average length of head with mandible: 1.32 mm Average length of head without mandible: 0.9 mm Width of head: 0.93 mm Overall length: 4.92 mm	
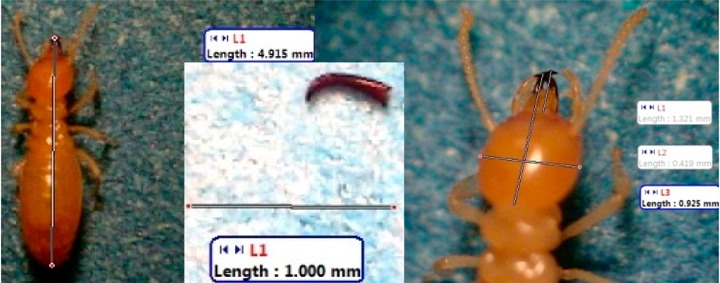
C8	*Microtermes insperatus* Characteristics: Average length of head with mandible: 1.53 mm Average length of head without mandible: 1.09 mm Width of head: 0.95 mm Overall length: 5.48 mm	
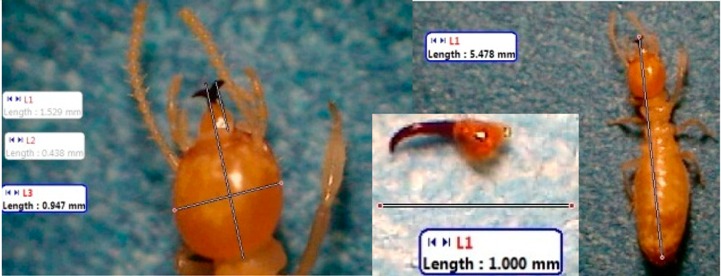
C9	*Microtermes insperatus* Characteristics: Average length of head with mandible: 1.13 mm Average length of head without mandible: 0.82 mm Width of head: 0.79 mm Overall length: 3.43 mm	
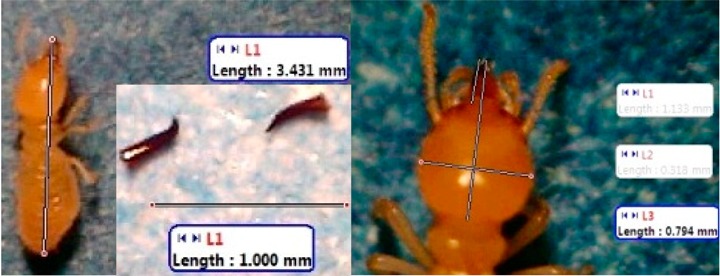
C10	*Microtermes insperatus* Characteristics: Average length of head with mandible: 1.46 mm Average length of head without mandible: 1.03 mm Width of head: 0.91 mm Overall length: 5.45 mm	
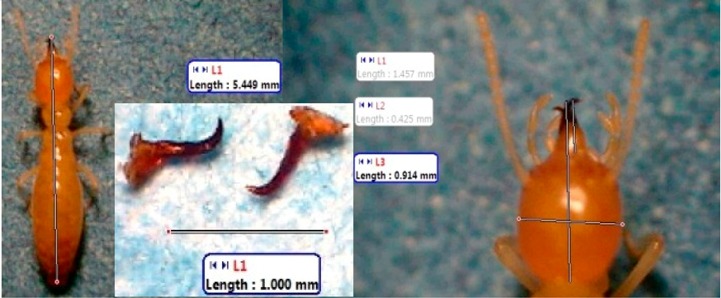
C11	*Microtermes insperatus* Characteristics: Average length of head with mandible: 1.21 mm Average length of head without mandible: 0.93 mm Width of head: 0.85 mm Overall length: 4.48 mm	
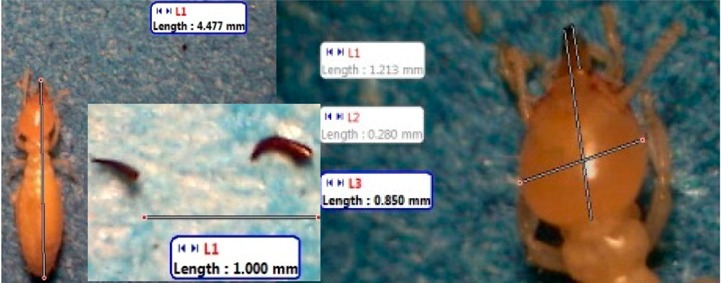
D1	*Microtermes insperatus* Characteristics: Average length of head with mandible: 1.18 mm Average length of head without mandible: 0.91 mm Width of head: 0.79 mm Overall length: 3.28 mm	
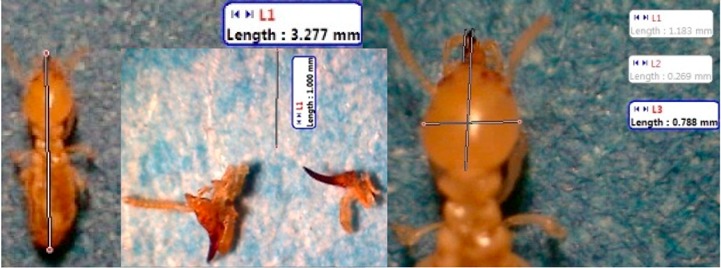
D2	*Microtermes insperatus* Characteristics: Average length of head with mandible: 1.4 mm Average length of head without mandible: 1.02 mm Width of head: 0.9 mm Overall length: 4.21 mm	

## References

[1] Robinson W.H. (1996). Termites. Urban Entomology, Insect and Mite Pests in the Human Environment.

[2] Nandika D. Satu Abad Perang Melawan Rayap. Proceedings of the Workshop Mitigasi Bahaya Serangan Rayap Pada Bangunan Gedung.

[3] Tsunoda K., Herliyana E.N., Hadi Y.S. (2012). Termite-susceptible species of wood for inclusion as a reference in Indonesian standardized laboratory testing. Insects.

[4] American Society for Testing and Materials (ASTM) (2008). ASTM D 1758-08 Standards Test Method of Evaluating Wood Preservatives by Field Tests with Stakes.

[5] Ahmad M. (1958). Key to the Indi-Malayan termites. Biologia.

[6] Tho Y.P. (1992). Termites of Peninsular Malaysia.

[7] Arinana, Haneda N.F., Nandika D., Prawitasari W.A. Damage intensity of house building and termite diversity in Perumahan Nasional Bumi Bekasi Baru, Rawalumbu Bekasi. *The Utilization of Biomass from Forest and Plantation For Environment Conservation Efforts*. Proceedings of the 6th International Symposium of Indonesian Wood Research Society.

[8] Eggleton P., Bignell D.E., Roisin Y., Lo N. (2011). An introduction to termites: Biology, taxonomy and functional morphology. Biology of Termites: A Modern Synthesis.

[9] Wang C., Powel J.E., Scheffrahn R.H. (2003). Abundance and distribution of subterranean termites in Southern Mississippi Forests (Isoptera: Rhinotermitidae). Sociobiology.

[10] Tarumingkeng R.C. (2000). Manajemen Deteriorasi Hasil Hutan.

[11] Rilatupa J. (2006). Kondisi komponen konstruksi bangunan tinggi dan hubungannya dengan karakteristik serangan rayap. J. Sains Teknol. EMAS.

[12] Mahaney W.C., Zippin J., Milner M.W., Sanmugadas K., Kancock R.G.V., Aufreiter S. (1999). Chemistry, mineralogy and microbiology of termite mound soil eaten by chimpanzees of the Mahal mountains, Western Tanzania. J. Trop. Ecol..

[13] Hillel D. (2004). Introduction to Environmental Soil Physics.

[14] White R.E. (2006). Principles and Practice of Soil Sciences Fourth Edition.

[15] Lee K.E., Wood T.G. (1971). Termite and Soil.

[16] Foth H.D. (1994). Dasar-Dasar Ilmu Tanah.

[17] Nandika D., Rismayadi Y., Diba F., Mubin N. (2015). RAYAP Biologi dan Pengendaliannya.

[18] Bahtiar E.T., Nugroho N., Karlinasari L., Surjokusumo S. (2014). Human comfort period inside and outside bamboo stands. J. Environ. Sci. Technol..

[19] Axelsson E.P., Andersson J.A. (2012). Case Study of Termite Mound Occurance in Relation to Forest Edge and Canopy Cover within the Barandabhar Forest Corridor in Nepal. Int. J. Biodivers. Conserv..

